# Large impact of the apoplast on somatic embryogenesis in *Cyclamen persicum *offers possibilities for improved developmental control *in vitro*

**DOI:** 10.1186/1471-2229-10-77

**Published:** 2010-04-28

**Authors:** Claudia Hoenemann, Sandra Richardt, Katja Krüger, Andreas D Zimmer, Annette Hohe, Stefan A Rensing

**Affiliations:** 1 Leibniz-Institute of Vegetable and Ornamental Crops (IGZ), Department Plant Propagation, Kuehnhaeuser Strasse 101, 99189 Erfurt, Germany; 2 University of Freiburg, Faculty of Biology, Hauptstrasse 1, 79104 Freiburg, Germany; 3 QIAGEN GmbH, Qiagenstrasse 1, D-40724 Hilden, Germany; 4 University of Freiburg, Faculty of Biology, Plant Biotechnology, Schaenzlestrasse 1, 79104 Freiburg, Germany

## Abstract

**Background:**

Clonal propagation is highly desired especially for valuable horticultural crops. The method with the potentially highest multiplication rate is regeneration via somatic embryogenesis. However, this mode of propagation is often hampered by the occurrence of developmental aberrations and non-embryogenic callus. Therefore, the developmental process of somatic embryogenesis was analysed in the ornamental crop *Cyclamen persicum *by expression profiling, comparing different developmental stages of embryogenic cell cultures, zygotic vs. somatic embryos and embryogenic vs. non-embryogenic cell cultures.

**Results:**

The analysis was based on a cDNA microarray representing 1,216 transcripts and was exemplarily validated by realtime PCR. For this purpose relative transcript abundances of homologues of a putative receptor kinase, two different glutathione S-transferases (GST), a xyloglucan endotransglycosylase (XET) and a peroxidase (POX) were quantitatively measured by realtime PCR for three different comparisons. In total, 417 genes were found to be differentially expressed. Gene Ontology annotation revealed that transcripts coding for enzymes that are active in the extracellular compartment (apoplast) were significantly overrepresented in several comparisons. The expression profiling results are underpinned by thorough histological analyses of somatic and zygotic embryos.

**Conclusions:**

The putative underlying physiological processes are discussed and hypotheses on improvement of the protocol for *in vitro *somatic embryogenesis in *Cyclamen persicum *are deduced. A set of physiological markers is proposed for efficient molecular control of the process of somatic embryogenesis in *C. persicum*. The general suitability of expression profiling for the development and improvement of micropropagation methods is discussed.

## Background

Plant micropropagation on a commercial scale has developed since the 1960s and gained high impact during the last centuries for clonal mass propagation especially of ornamental crops [1,2]. The method with the potentially highest multiplication rate is regeneration via somatic embryogenesis (s.e.), which was initially described in 1958 for *Daucus carota *[3,4]. Since then, somatic embryogenesis systems have been developed for a multitude of plant species, but despite the large number of published protocols, only very few systems are actually used in commercial plant propagation. This can be put down to the fact that many protocols are inadequately reproducible, a differing fraction of the embryos shows developmental aberrations and non-embryogenic callus frequently arises during the use of indirect embryogenesis systems. Due to the often insufficient reproducibility, these problems are difficult to solve by empirical protocol changes. Yet, efficient propagation by somatic embryogenesis would be the method of choice for plant species that do not allow clonal propagation by cuttings, including the ornamental crop *Cyclamen persicum*.

In the last decade a series of genes have been identified that play a role in the s.e. of seed plants (for review see e. g. [5,6]). The expression of single genes has frequently been investigated in the course of somatic and zygotic embryogenesis and the importance of certain gene products has been proven for individual stages of development in different plant species. Developmental aberrations, however, can rarely be attributed to single or few genes in the course of s.e. Instead, it can be assumed that the whole expression pattern is changed during the course of the culture. Thus, in problem-oriented approaches, microarray-based expression analyses might give a more complete picture of the cultures' physiology that subsequently allows molecular physiologically founded progression of propagation protocol development.

During the last five years a steadily increasing number of studies has been published, analysing the process of somatic embryogenesis by gene expression profiling (e.g. in *Glycine max*: [7], *Picea abies*: [8], *Oryza sativa*: [9]; *Zea mays*: [10]; *Gossypium hirsutum*: [11,12], *Cichorium intybus*: [13], *Triticum aestivum*: [14], *Elaeis guineensis*: [15]). However, only a few studies aimed at an improvement of the protocol for mass propagation. In this context Stasolla et al. [16,17] have been the first to establish a connection between gene expression studies in s.e. and application-oriented work on protocol development and optimisation by analysing gene expression patterns in response to medium supplementation for improvement of maturation of somatic embryos of *Pinus glauca*.

S.e. in *Cyclamen persicum *represents a well established system very much resembling that in *D. carota *[18]. In contrast to *D. carota*, an efficient clonal propagation method for *C. persicum *is highly desired in the horticultural industry. Following publication of the original protocol, the system was developed further by establishing suspension and bioreactor cultures [19-22] and developing methods for desiccation and cryoconservation of the somatic embryos [23,24]. However, major problems caused by development of non-embryogenic cell lines, absence of a maturation phase and occurrence of malformed embryos could not be solved to date [25,26].

Recently, two proteomic studies have been conducted to analyse the process of s.e. in *C. persicum*. Winkelmann et al. [27] compared the proteome of somatic and zygotic embryos of *C. persicum*, whereas Lyngved et al. [28] analysed embryogenic and non-embryogenic callus before induction of somatic embryo development. Both studies were of fundamental character, not primarily aiming at improvement of the *in vitro *culture method. Therefore, we conducted an expression profiling study based on a cDNA microarray representing 1,216 transcripts identified in a preceding EST analysis using a normalised cDNA library prepared from embryogenic cell cultures and young somatic embryos [29]. Thus, in contrast to the proteomic studies, our expression analyses were restricted to a group of pre-selected genes expressed during embryogenesis. Due to the normalisation process also low expressed signalling genes were included. The overall goal of the present study was to identify key physiological pathways that are (i) fundamentally involved in s.e. in *C. persicum *(ii) prone to cause aberrant development and (iii) accessible for manipulation by *in vitro *culture. Therefore, the microarray was hybridised with cDNA generated from a selection of different embryogenic and non-embryogenic cell cultures as well as from zygotic embryos. These data were evaluated with the aim of generating new hypotheses for improving the micropropagation protocol using the expression of specific genes as physiological markers. A more general goal of our study was to prove the suitability of expression profiling analyses as a molecular physiologically based approach for development and improvement of to date mainly empiric *in vitro *culture methods.

## Results and Discussion

### Global expression profiling results

We analysed the expression of 1,216 transcripts (Additional file 1) during somatic and zygotic embryogenesis in *C. persicum *using a cDNA microarray derived from annotated transcripts of a previous analysis [29]. The overall aim of our study was to develop new hypotheses to improve the protocol of s.e. Therefore, we analysed gene expression during different stages of induction and development of embryogenic cell cultures as well as in mature somatic and zygotic embryos.

**Figure 1:**
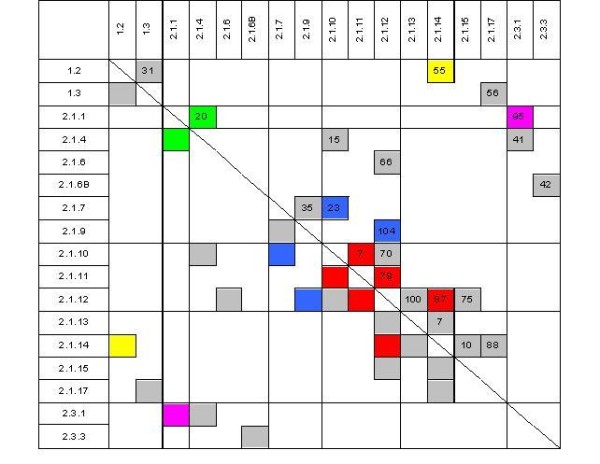
**Overview of microarray analyses performed**. Tissues are specified by their IDs (see table 2 for details). Numbers specify the amount of differentially expressed genes in an experiment (p ≤ 0.005). Those experiments that are discussed in detail are marked with colours according to the experimental question: development of somatic embryos (red), putative reasons for developmental arrest in the globular stage (blue), comparison of embryogenic and non-embryogenic cell cultures (magenta), comparison of somatic and zygotic embryos (yellow) as well as comparison of a diploid and a tetraploid callus line (green).

**Figure 2:**
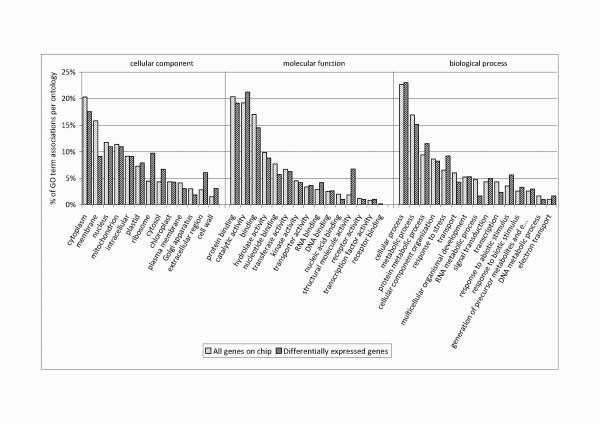
**GO annotation**. GO annotation of all transcripts present on the microarray in comparison to the differentially expressed genes in the eight selected experiments. The annotation is shown in percentage of GO associations per gene set and GO category.

In total, 417 genes were found to be differentially expressed in 21 experiments comparing 17 different tissues or conditions (p ≤ 0.005) (Additional file 1). After pairwise analysis (Figure 1) we selected eight experiments comparing ten different tissues for detailed interpretation (Additional file 2). These comparisons have been selected since they allow to draw interesting conclusions about the process of s.e. and provide indications for the improvement of propagation protocols. Within this reduced set of experiments a total of 279 genes were found to be differentially expressed. These comparisons were evaluated with regard to different questions as given in Figure 1, i.e. development of the somatic embryos (marked in red), putative reasons for developmental arrest in the globular stage (marked in blue), difference between embryogenic and non-embryogenic cell cultures (marked in magenta), difference between somatic and zygotic embryos (marked in yellow) as well as of the difference between a diploid and a tetraploid callus line (marked in green). In order to find key physiological pathways that are (i) fundamentally involved in s.e. in *C. persicum *(ii) prone to cause aberrant development and (iii) accessible for manipulation by *in vitro *culture, we subjected our data to Gene Ontology (GO) annotation [30] (Figure 2). It was tested which GO terms were significantly over- or underrepresented among the 279 differentially expressed genes as compared to the complete set of genes on the chip. From the summary of these analyses (Table 1) it can be deduced that predominantly processes of stress response located in the apoplast are important for s.e. in *C. persicum*. Therefore, these are shown and discussed in detail in the following paragraphs.

**Table 1:** Gene Ontology terms significantly overrepresented among the differentially expressed genes comparing tissue 1 with tissue 2 (Fisher's exact test, p ≤ 0.05)

Comparison		Gene Ontology		
**Tissue 1**	**Tissue 2**	**Biological Process**	**Cellular Component**	**Molecular Function**

zygotic embryo (ID 1.3)	somatic embryo (ID 2.1.14)		Chloroplast	

embryogenic callus (ID 2.1.1)	non-embryogenic callus (ID 2.3.1)	response to stress, response to biotic stimulus	extracellular region	

embryogenic tissue, 4 h after induction (ID 2.1.11)	embryogenic tissue, 3 d after induction (ID 2.1.12)		cell wall	catalytic activity

embryogenic tissue, 3 d after induction (no torpedo-shaped embryos generated) (ID 2.1.9)	embryogenic tissue, 3 d after induction (torpedo-shaped embryos generated) (ID 2.1.12)	response to abiotic stimulus	cell wall, cytosol	

embryogenic tissue, 3 d after induction (ID 2.1.12)	Torpedo-shaped somatic embryos, 3 weeks after induction (ID 2.1.14)	response to abiotic stimulus		

**Figure 3:**
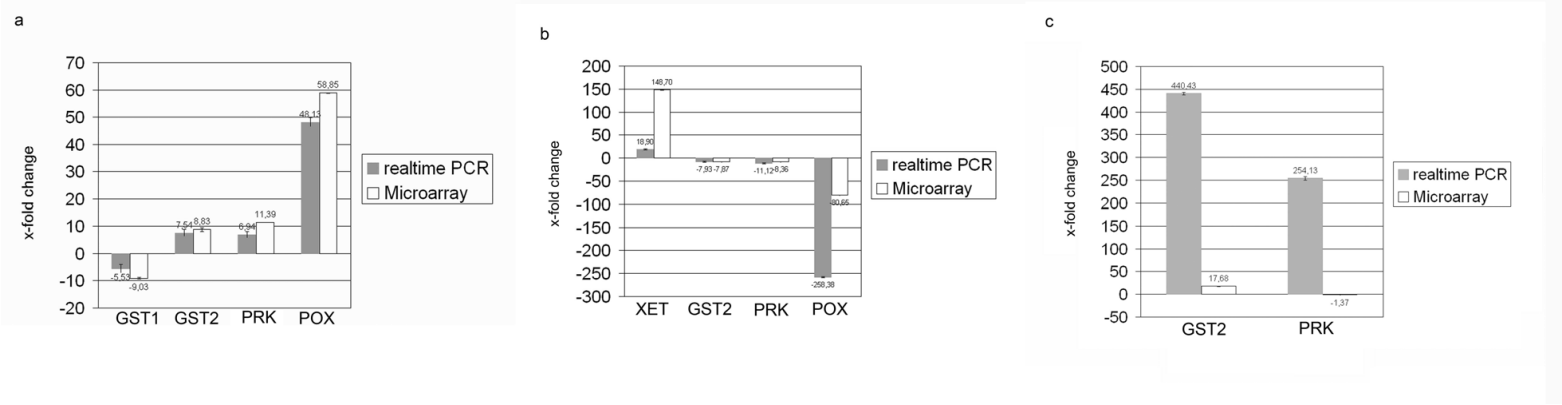
**Validation of microarray results using realtime PCR (arithmetic mean of fold change +/- average absolute deviation (AAD))**. a: GST1 (CYC01T7_E12), GST2 (CYC32T7_B11), putative receptor kinase (PRK) (CYC04T7_G06) and POX (CYC04T7_G04) in the comparison embryogenic suspension 4 h (2.1.11) vs. 3 d after induction (2.1.12). b: XET (CP_59_C1), GST2 (CYC32T7_B11), putative receptor kinase (PRK) (CYC04T7_G06) and POX (CYC04T7_G04) in the comparison embryogenic suspension 3 d after induction (2.1.12) vs. selected torpedo-shaped somatic embryos 3 weeks after induction (2.1.14). c: GST2 (CYC32T7_B11) and putative receptor kinase (PRK) (CYC04T7_G06) in the comparison: zygotic embryos (1.2) vs. selected torpedo-shaped somatic embryos 3 weeks after induction (2.1.14)

**Figure 4:**
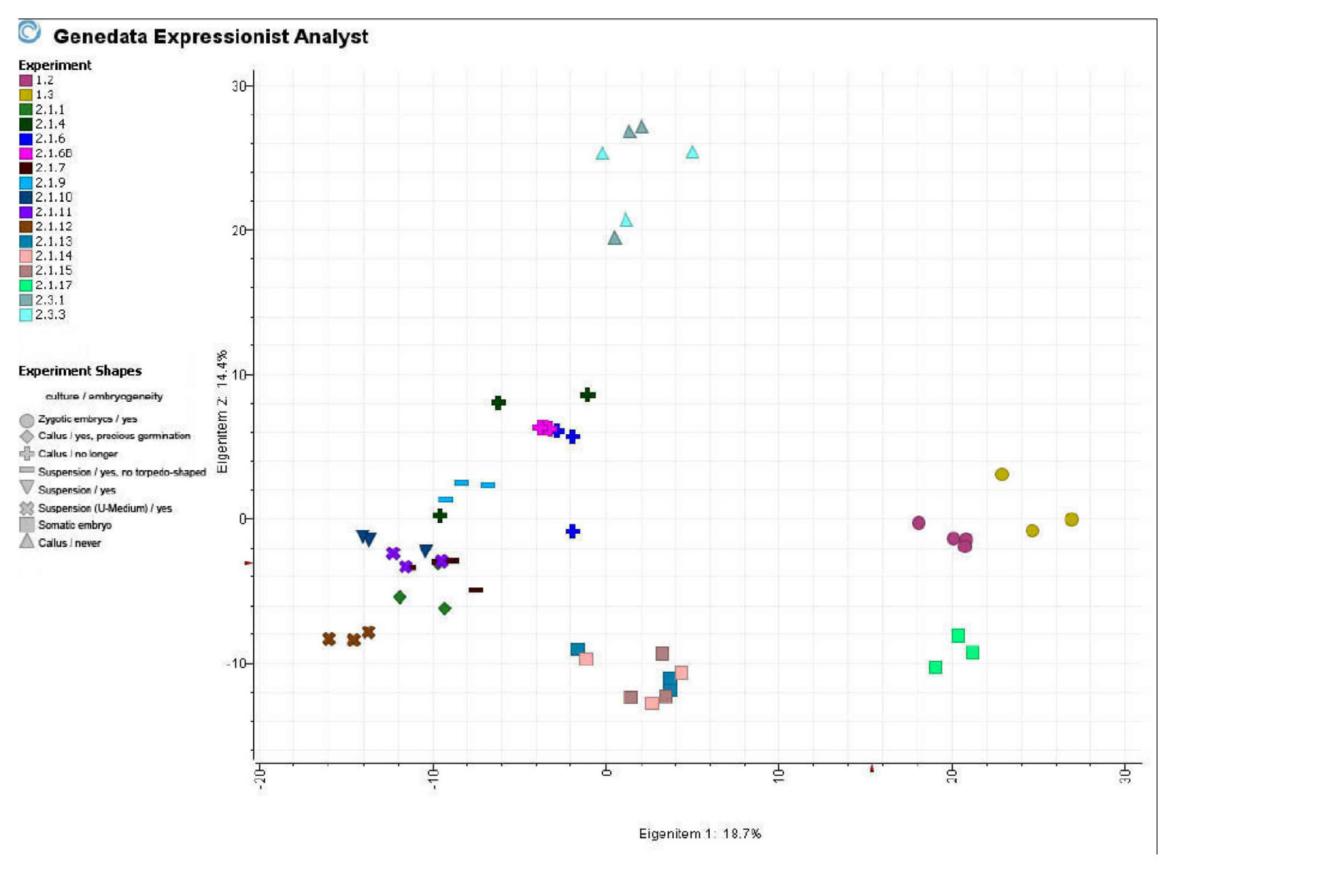
**PCA showing differences in gene expression for the total of 17 tissues under different conditions**. Differences between treatments as well as high reproducibility of the biological replicates are demonstrated. Tissues not interpreted in detail are marked in grey, all tissues selected for detailed analysis are coloured. (1.2: zygotic embryos, 2.1.1: embryogenic callus (diploid), 2.1.4: embryogenic callus (tetraploid) 2.1.7: embryogenic suspension (blocked in the globular stage) before induction, 2.1.9 embryogenic suspension (blocked in the globular stage) 3 d after induction, 2.1.10: embryogenic suspension before induction, 2.1.11: embryogenic suspension 4 h after induction, 2.1.12: embryogenic suspension 3 d after induction, 2.1.14: selected torpedo-shaped embryos 3 weeks after induction, 2.3.1: non-embryogenic callus before induction)

In order to confirm the microarray data, the expression of ten randomly chosen differentially expressed genes was validated by realtime PCR. Here, the qualitative results of the microarray were proven in nine out of the ten transcripts, moreover for eight transcripts the results of the two methods corresponded as well quantitatively (Figure 3). Therefore, we regard the microarray data as generally reliable. A principal component analysis (PCA) demonstrates the high reproducibility of the three independent biological replicates (Figure 4). The first two Eigen items contribute 18.7 and 14.4% of the variance, respectively, and are sufficient to separate the data according to the broad developmental stage of the tissues (Figure 4). Additional Eigen items result in an ever better resolution of developmental stages (data not shown). The PCA results nicely reflect the tissue/experiment selection described above.

### Development of somatic embryos

#### Three days after induction

The total number of genes differentially expressed when comparing cells at induction and later developmental stages increased with ongoing periods of development (Figure 1: red marked comparisons). Four hours after induction only seven genes were differentially expressed as compared to the cells prior to induction. In contrast, three days later 79 genes showed differential expression as compared to four hours after induction. Two out of these 79 genes encode homologues of a chitinase (CYC12T7_E02) and a peroxidase (POX) (CYC04T7_G04) and belong to the GO term "cellular component"/"cell wall" that was overrepresented in this comparison (Table 1). A second POX (CYC11T7_B02) homologue was regulated similarly, although not annotated within this GO category. All three genes were up-regulated three days after induction as compared to four hours after transfer to growth regulator free medium.

**Figure 5:**
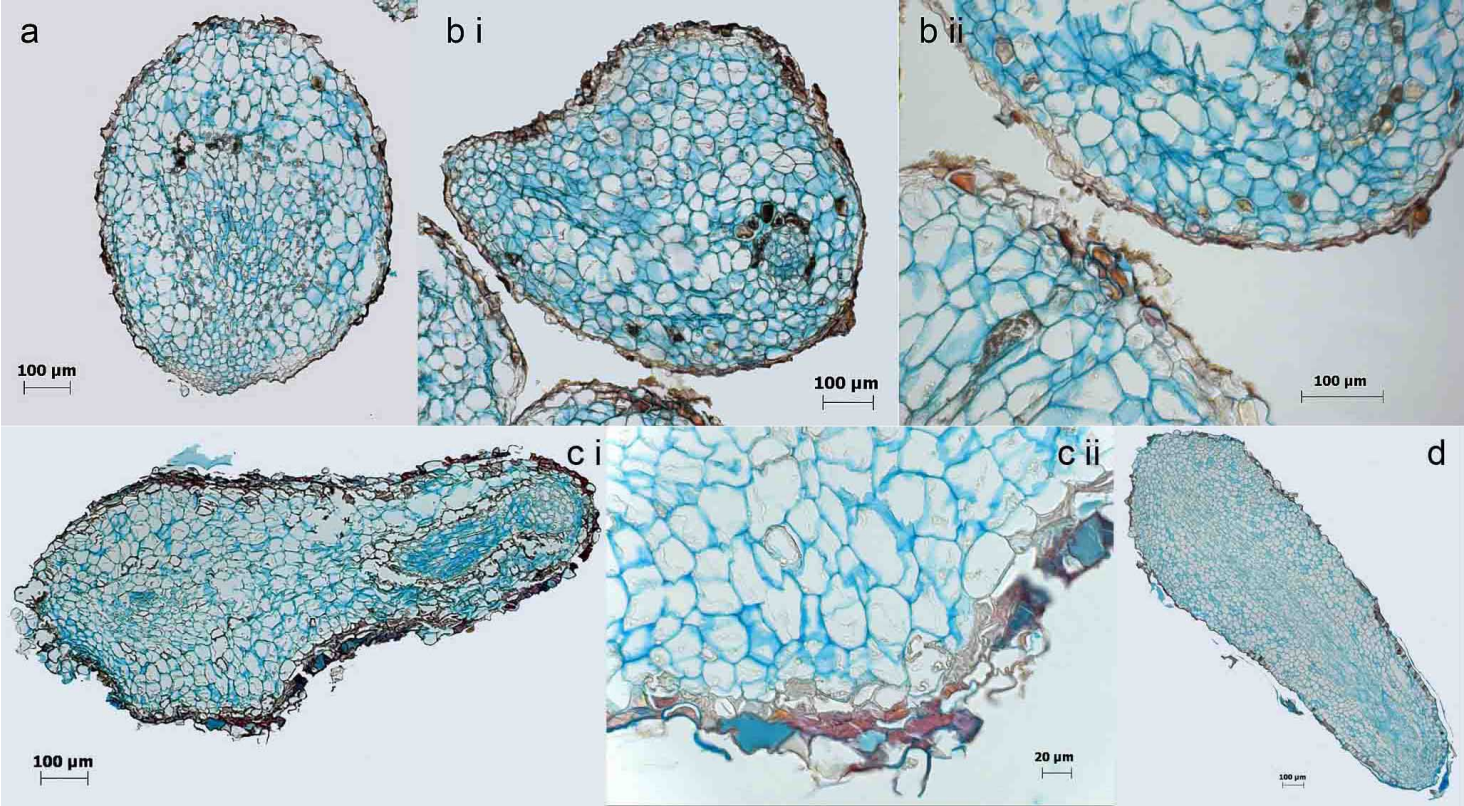
**Histological analyses of different stages of somatic embryos 3 weeks after induction by transfer of the cells to growth regulator free medium using FCA staining**. Cell walls are stained in blue, lignified or cutinised cell walls in orange to brownish red and nuclei in light purple. Globular shaped embryos display an irregular anatomical organization (a and b). Torpedo-shaped embryos, although displaying a regular outer shape, can be anatomically abnormal (c). Orange stained cell walls are found within globular shaped embryos (a and b). A rough and irregular epidermis structure is typical for somatic embryos (b ii and c ii). However, morphologically and anatomically normal somatic embryos also have been detected (d).

It has been found that s.e. in *C. persicum *resembles that of *D. carota *in terms of transcripts involved [29]. In *D. carota*, a mutant cell line has been identified in which somatic embryo development is arrested in the pre-globular stage due to incorrect protoderm development [31]. This line morphologically resembles the somatic embryos in our study that - although partially developing beyond the (pre)-globular stage - displayed an aberrant epidermal cell layer (Figure 5b ii and 5c ii). De Jong et al. [31] were able to rescue the cell cultures by addition of an endochitinase. From this they deduce an essential role of the endochitinase in formation of a proper protoderm in the pre-globular stage that in turn is a prerequisite for transition to subsequent embryo stages. The expression of a chitinase in induced embryogenic cultures in our study supports this hypothesis. Arabinogalactan proteins, known to be active compounds in so-called conditioned culture medium, have been suggested to be the substrate of this chitinase in *D. carota *[32].

In addition, a cationic peroxidase (POX) has been identified as being essential for pre-globular somatic embryo development for the first time in *D. carota *[33]. This corresponds to our results showing up-regulation of a POX homologue (CYC04T7_G04) three days after induction. Cordewener et al. [33] concluded from their results that cationic POX activity prevented cell size expansion, thus causing development of small cytoplasm-rich cells as a prerequisite for embryo development. Takeda et al. [34] analysed the expression and function of a cell wall-bound cationic POX during somatic embryogenesis in *Asparagus officinalis*. In this study, transcripts of putative POX genes were also most abundant during early s.e. and depletion of lignin precursors is discussed as a possible function in cell differentiation.

In this comparison 27 transcripts were found to be up-regulated between four hours and three days after induction and repressed again three weeks after induction as compared to the expression level three days after transfer to growth regulator-free medium (Additional file 2). Thus, these genes seem to be specific for induction and early developmental processes. One of those encodes a homologue of a glutathione S-transferase (GST) (CYC32T7_B11). GSTs exist in many isoforms in plants and are active in cellular detoxification processes, in which the different isoforms are specific for different substrates [35]. Since two transcripts of cytochrome P450 homologues (CYC14T7_A10 and CYC32T7_D03) were also highly abundant specifically three days after induction, cellular detoxification seems to be important for this early developmental process. Whereas other authors deduce a central role of GST in the regulation of somatic embryogenesis from its auxin-inducibility [14], the GST homologue in our study was in fact up-regulated in response to auxin removal. However, two out of the five GST homologues (CYC01T7_E12 and CYC33T7_F07) in our study were repressed upon auxin-removal, whereas the remaining two (CYC16T7_B04 and CYC29T7_E07) did not show differential expression when the cells were transferred to auxin-free medium. This is in line with results from Pan et al. [36] demonstrating differential regulation of three GST homologues during somatic embryogenesis of *Citrus sinensis*. These authors argue that GST, together with other enzymes involved in oxidative stress reactions, might play a role in regulation of redox changes critical for triggering s.e.

#### Three weeks after induction

Three weeks after induction torpedo-shaped somatic embryos were present in the culture. Here, 87 genes were differentially expressed as compared to the cultures three days after induction. Two of the genes specifically up-regulated at this stage encode homologues of a xyloglucan endotransglycosylase (XET, an enzyme synthesising or hydrolysing xyloglucans) (CYC16T7_D07 and CYC32T7_F01). Xyloglucans are common cell wall compounds and their regulation has thus also been analysed in the context of somatic embryogenesis [37]. In addition, they also serve as cell wall-bound seed storage compounds as in the case of members of the Primulaceae [38] and also in *C. persicum *[39]. Winkelmann et al. [27] observed high abundance of XET in the endosperm of *C. persicum *seeds in a proteomic study comparing somatic and zygotic embryos. In contrast, our study showed a high abundance of XET homologue transcripts in three week old somatic embryos as compared to cultures three days after induction. Likewise, one of the XET homologues (CYC32T7_F01) was also up-regulated in somatic embryos as compared to their zygotic counterparts. Thus, somatic embryos might accumulate storage compounds that are confined to the endosperm in zygotic embryogenesis. It remains to be investigated whether this is a prerequisite for successful development of somatic embryos (that necessarily lack an endosperm) or whether this might be a reason for abnormal development of somatic embryos and can be avoided by changes in growth media composition.

### Differences between cell lines

#### Developmental arrest in the pre-torpedo stage

In the current study we also compared two cell cultures that differed in their ability to develop torpedo-shaped embryos (Figure 1: marked in blue). The culture in standard medium was developmentally arrested in the globular stage and lost its ability to form torpedo-shaped embryos after the 75^th ^subculture. In contrast, when the same culture was transferred to the richly supplemented U-medium, globular embryos developed further into torpedo-shaped embryos. These two cultures differed in gene expression before induction with regard to only 23 genes. However, three days after induction, 104 genes were found to be differentially expressed, which is the highest number of differentially expressed genes in any pairwise comparison of our study. At this time the deviating phenotype (development of torpedo-shaped embryos 14 d after induction) was not yet visible. Thus, while some of the differences might be due to the differing media, the differential expression of some of these genes might be causal for the developmental arrest, resp. developmental progression. Interestingly, genes belonging to the GO term "cellular component"/"cell wall" again were found to be significantly overrepresented among the genes differentially expressed in this comparison (Table 1). Among these were the two early responding POX (CYC04T7_G04) and chitinase (CYC12T7_E02) transcripts, homologues of which have been identified in *D. carota *as causing developmental arrest [33,31]. In our study both were induced in the suspension culture that later on developed torpedo-shaped embryos as compared to the culture arrested in the globular stage. Since our culture was rescued by transfer to richly supplemented U-medium, we assume one of the supplements as a necessary factor. As our cultures lost their ability to develop torpedo-shaped embryos over time, one might hypothesize that this was caused by depletion of a factor that was not present in sufficient concentration in the standard medium, e.g. microelements that serve as co-factors of stress-related enzymes.

#### Embryogenic and non-embryogenic cell cultures

In the comparison of gene expression between an embryogenic and a non-embryogenic cell line before induction, 95 genes were found to be differentially expressed (Figure 1: marked in magenta). As found within the comparison of suspension cultures developing torpedo-shaped embryos vs. being arrested in the pre-torpedo stage, the differential expression pattern was eminent before the differing phenotypes were realised. Thus, part of the 95 genes might be regarded as causal for the ability of s.e. and not just correlative with the phenotype. However, one has to keep in mind that these two cell lines were of similar but not of identical genotype, since they have been established from different plant individuals and were of different age.

Regarding the GO category "cellular component", again the term "extracellular region" was significantly overrepresented among the genes that were differentially expressed in this comparison (Table 1). This is in line with research on somatic embryogenesis in other species, showing that the extracellular matrix is an active component of signal transduction [40-42]. In our study, one of the genes of the corresponding ontologies, that was found to be up-regulated in embryogenic callus, was again the chitinase homologue (CYC12T7_E02) discussed above, which once more supports the relevance of this enzyme in early somatic embryogenesis that has been reported e.g. for *D. carota *[31,32]*Picea glauca *[43] and *Pinus caribea *[44]. Besides chitinase, also a group of three genes encoding homologues of pectin modifying enzymes (Pectinesterase: CYC26T7_G03, Pectate lyase: CYC26T7_E05 and CYC26T7_E10) belonging to the GO term "extracellular region" as well, were up-regulated in embryogenic as compared to non-embryogenic callus. Also, a pectinacetylesterase (CYC14T7_B05) as a fourth enzyme within this group showed a similar expression pattern, yet this enzyme was not annotated within the "extracellular region" GO category. Pectins are major components of the middle lamella and important for intercellular adhesion. In this context, it has already been shown that the inhibition of the transport of pectins to the cell wall caused morphological embryo defects in zygotic embryos of *A. thaliana *[45]. Bouton et al. [46] described two allelic *A. thaliana *mutants (qua1-1 and qua1-2) that carry T-DNA insertions in a gene encoding a putative glycosyltransferase that is involved in biosynthesis of pectic polysaccharides. These mutants also show reduced cell adhesion and a dwarf phenotype. Likewise, in *Nicotiana plumbaginifolia *a mutant (nolac-H18) deficient in a glycosyltransferase that is involved in pectin biosynthesis lost its ability to form tight intercellular attachments in callus cultures and also their ability to regenerate adventitious shoots [47]. Moreover, in some plant species embryogenic and non-embryogenic cell lines display differences in pectin composition, and it has been concluded that a pectin-conferred cell adhesion is a prerequisite for s.e. [48,49]. Verdeil et al. [50] showed that the acquisition of embryogenic competence in callus of *Cocos nucifera *was linked to the appearance of a fibrillar material containing pectin, coating the embryogenic cells. In our study we find homologues of genes encoding enzymes involved in pectin degradation and methylesterification to be up-regulated in the embryogenic cell line, which is less friable than the non-embryogenic one. This hints at pectin degradation and modification possibly being necessary for continuous remodelling of middle lamellas during active pre-embryogenic callus growth in *C. persicum*. In contrast, in the non-embryogenic cell line the much looser cell adhesion might be caused by reduced pectin content, which might be an important factor for the loss of embryogeneity due to reduced cell adhesion. Therefore, the *in vitro*-culture practice of selecting friable callus lines for better cultivation and establishment of suspension cultures [19] might in the case of *C. persicum *result in the selection of non-embryogenic cell lines. This hypothesis needs to be proven by detailed analyses of multiple embryogenic and non-embryogenic cell lines.

Another striking difference in gene expression between the embryogenic and the non-embryogenic cell line was the expression of three GST homologues, which were already discussed within the context of early s.e. Two were up-regulated in the embryogenic cell line (CYC01T7_E12 and CYC32T7_B11) in contrast to the non-embryogenic one, whereas a third one was repressed (CYC16T7_B04). This confirms the hypothesis that these GST homologues might be crucial for early somatic embryo development. One might also speculate on differences in auxin-responsiveness of these two cell lines, since auxin-dependent induction of GST-transcription has been reported in other plant s.e. systems [14]. In fact, in our study one of the GST homologues (CYC01T7_E12), that had been shown to be repressed upon auxin-removal, was also repressed in the non-embryogenic cell line (in contrast to the embryogenic one), although the latter cells were cultivated on auxin-containing medium.

Another differentially expressed gene (CYC14T7_H01) in this context that was specifically up-regulated in the embryogenic cell line belongs to the SERK family, which is also well known to be involved in s.e. in other plants. A SERK was the first gene specifically identified to be involved in s.e. [51]. Receptor protein kinases such as SERKs play a role in several signal transduction pathways that elicit a developmental response to exogenous input [52]. In *D. carota*, SERK was found to be expressed in embryogenic suspension cells and in early stages of both somatic and zygotic embryos [51]. Expression analyses of SERK1 in *Arabidopsis thaliana *revealed a slightly different expression pattern, since here the gene was already expressed during megasporogenesis and in late embryo vascular strands [53,54]. A correlation between SERK gene expression and somatic embryogenesis has been demonstrated for many other plant species in recent years, e.g. in *Helianthus annuus *[55], *Theobroma cacao *[56], *Oryza sativa *[57], or *Vitis vinifera *[58]. Out of the five genes in our study that are homologous to genes annotated as SERK [29], only one (CYC14T7_H01) was found to be differentially expressed in any of our experiments. This is in line with other studies showing that SERK expression is not restricted to the regulation of embryogenesis (either somatic or zygotic) but also plays a role in other developmental or physiological processes, e.g. adventitious shoot regeneration in *Helianthus annuus *[55] or host defence response in *Oryza sativa *[57,59].

Interestingly, in the non-embryogenic cell line, a putative argonaute (AGO) homologue (CYC33T7_C02) was significantly up-regulated that was not found to be differentially expressed in any other comparison. AGO proteins are part of the RNA-induced silencing complex (RISC) involved in posttranscriptional gene silencing via RNA interference (RNAi) [60]. Tahir et al. [61] have identified a gene of the AGO family in *Picea glauca *that proved to be essential for normal somatic embryo development. Similarly Takahata [62] has demonstrated differential expression of an AGO homologue during s.e. in *D. carota *and deduces that RNAi controlled gene expression is required for s.e. We regard our result as an indication that RNAi processes might also be involved in the common phenomenon of generation of embryogenic and non-embryogenic cell lines from identical explants or the loss of embryogenic competence in tissue culture.

### Comparison of zygotic and somatic embryogenesis

**Figure 6:**
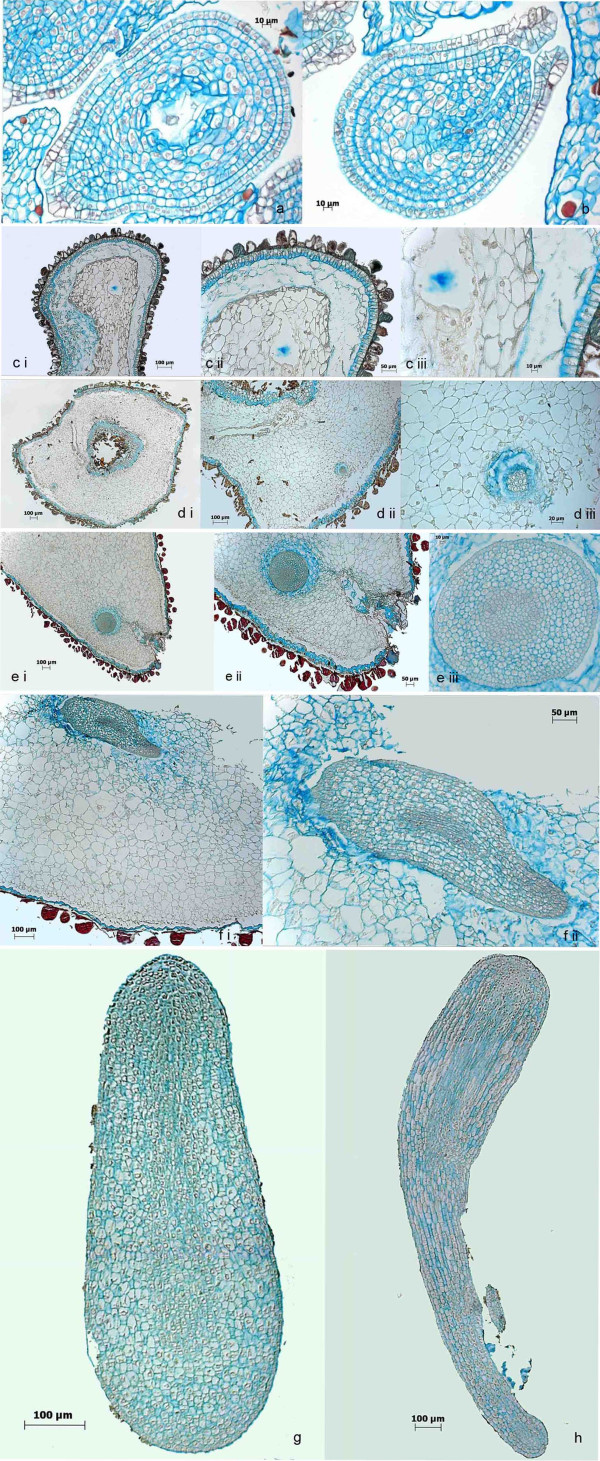
**Histological analyses of the development of zygotic embryos using FCA staining**. Cell walls are stained in blue, lignified or cutinised cell walls in orange to brownish red and nuclei in light purple. Zygotic embryo development starts within the ovules after pollination. Early stages (a: 0 d after pollination, b: 10 d after pollination) show ovules within the surrounding ovary tissue. The embryo sac and the micropyle are clearly visible. 30 d after pollination the endosperm becomes cellular and tiny multicellular embryos can be detected (c i to iii). 50 d after pollination globular shaped embryos have been formed (d i to iii). 60 d after pollination embryos become torpedo-shaped (transversal: e i to iii and longitudinal: f i to ii). 75 to 100 d after pollination zygotic (g and h, showing excised embryos) embryos reach their full size and maturation starts.

The anatomy of developing zygotic and somatic embryos is shown in Figures 5 and 6, respectively. Figure 6a and 6b show ovules before and ten days after pollination. The micropyle and the embryo sac can clearly be identified. Thirty days after pollination, the first tiny globular embryos were detected (Figure 6c). At this stage, the endosperm was at the transition stage to become cellular. Fifty days after pollination, embryos were in the globular stage (Figure 6d) and after 60 days a torpedo-shaped morphology developed (Figure 6e: transversal section, f: longitudinal section). Finally, 75-100 d after pollination the embryos reached their final size before maturation (Figure 6g and 6h). These periods of development are consistent with our former morphological analyses [25]. Most striking in the anatomical pictures presented here are the large size differences of zygotic embryo and endosperm cells: 423 +/- 61 μm^2 ^versus 1649 +/- 694 μm^2 ^(arithmetic mean +/- standard deviation).

In comparison, cells of somatic embryos in all developmental stages (Figure 5) are much larger (1484 +/- 508 μm^2^), comparable to the size of the cells of the endosperm.

In contrast to the strictly synchronised development of the zygotic embryos, three weeks after induction of the somatic embryos all stages were present simultaneously. Another striking difference between zygotic and somatic embryos appears to be the structure of the epidermis. Whereas zygotic embryos displayed a plain and unruffled outer cell layer (Figure 6g and 6h), the epidermis of somatic embryos was not that smooth (Figure 5b ii and 5c ii). As discussed above, abnormal formation of epidermal cell layers has been identified in *D. carota *as a reason for developmental arrest in the globular stage [31]. Likewise Tokuji and Kuriyama [63] identified malformation of the epidermis, and s.e. in the context of uniconazole (a gibberellic acid inhibitor) induced malformation of *D. carota *somatic embryos. Histological staining in our study also revealed the presence of cells with cutinised and/or lignified cell walls within globular-shaped embryos (Figure 5a and 5b) as well as in the epidermis of torpedo-shaped embryos (Figure 5c). This is another hint on the impact of the extracellular matrix on somatic embryo induction and development postulated also in other studies [40-42].

These differences in the anatomy of somatic and zygotic embryos of *C. persicum *were corroborated by the corresponding gene expression analyses (Figure 1: marked in yellow). Since in this comparison, diploid (zygotic) and tetraploid (somatic) material was compared, we interpret only those data that were not among the homologues of genes differentially expressed in a comparison of diploid and tetraploid callus (Figure 1: marked in green). Three of the transcripts that were highly abundant specifically in somatic embryos are involved in oxidative stress response (homologues of GST (CYC32T7_B11), superoxide dismutase (CYC25T7_A12) and catalase (CYC13T7_H06)). As discussed above, up-regulation of genes involved in oxidative stress response is a typical reaction for early s.e. However, in our study, this comparison has been made three weeks after induction, using selected torpedo-shaped embryos. Therefore, we interpret these data as a hint that the signals inducing embryogenesis might linger, so that the cultures are prone to undergo secondary embryogenesis (which is in fact often observed in our cultures). From these data we deduce a necessity to more effectively remove auxins from the culture upon induction. This might be realised by supplementation of the medium with activated charcoal or even auxin inhibitors.

## Conclusions

From the expression profiling data presented here, we can determine differentially expressed genes that are intermediate and later responders of the developmental process under investigation. Therefore, the data give valuable insights and constitute a basis for new hypotheses on how the process of s.e. in *C. persicum *might be improved *in vitro*:

1. During cell line selection more attention should be paid to cell adhesion, since this might be a factor promoting s.e. in *C. persicum*. Detailed analyses using different embryogenic and non-embryogenic cell lines are planned for the future in order to generate reliable data on the impact of pectin-mediated cell adhesion. To support proper cell line selection, the use of expression profiling of genes involved in pectin degradation and remodelling as a physiological marker will be tested.

2. High expression levels of AGO, GST and SERK homologues are additional putative indicators for the identification of embryogenic vs. non-embryogenic callus, respectively. This will be validated by screening of different cell lines under inductive and non-inductive conditions. A possible involvement of RNAi in the loss of embryogenic competence will be investigated as well.

3. Proper epidermis formation in early embryonic stages might be a prerequisite to avoid embryo malformation. Chitinase and POX activity will be checked for suitability as a physiological marker.

4. During *in vitro *culture more attention should be paid to buffering or controlling the pH as a means to influence redox homeostasis and specific enzyme activity. Putative differential expression of GST, POX, and chitinase homologues are of special interest in this context.

5. The effect of richly supplemented U-medium on overcoming the developmental arrest at the globular embryo stage might be analysed by studying expression of the early responding genes GST, POX and chitinase in response to supplementation of the standard medium with individual compounds of the U-medium.

6. The effect of rich carbohydrate supplementation of the medium - a technique well known from other plant species to promote embryo maturation - on cell size and expression of XET in somatic embryos will be analysed in order to check whether incorrect storage compound accumulation might cause malformation of embryos.

7. Secondary embryogenesis might be another major reason for formation of aberrant embryos. Therefore, protocol changes should aim at preventing the auxin signal to linger after auxin removal. This might be achieved by addition of activated charcoal. Moreover, antagonistic signals might be given by addition of gibberellic acid or even auxin inhibitors upon auxin removal.

Thus, we were able to develop new hypotheses on *in vitro *protocol improvement based on the molecular physiological knowledge we gained by gene expression profiling. Future work will focus on in-depth analyses of these hypotheses.

The following limitations of this work should be taken into account when interpreting the data:

1. Different genotypes of the cell lines could add bias to comparative expression profiling.

2. Detection limit of cDNA microarray analysis could prevent some expression changes to be detected. The realtime PCR analysis showed that at least for some genes the sensitivity of the cDNA microarray to detect differential expression was lower in comparison.

3. The mixture of cell types of some analysed tissues could limit sensitivity as well, since small expression changes in only single cell types would be masked.

4. The fragmentary nature of the EST sequences might limit their correct annotation.

5. Only about 4% of the transcripts have been analyzed by microarray analysis.

However, since the suggested protocol improvements rely on a much more detailed physiological knowledge than the common development process of propagation protocols, we expect this strategy to be more effective in terms of time and reliability. Novel cost-effective sequencing technologies will also enable the expansion of the current studies to the complete transcriptome of *C. persicum*.

## Methods

### Tissue culture

The cell lines of the genotype "3" were established as described by Schwenkel and Winkelmann [18] from unfertilised ovules of a single plant from the cultivar 'Sierra Purple Flame' in May 2003 and a second time in August 2005. During continuous propagation, different subtypes developed as described in detail in Table 2. Cell line "12G" was established in March 1991 from the cultivar 'Purple Flamed' (genotype 3738, [64]), which was non-embryogenic from the beginning. The cell lines were cultivated on MS based growth regulator-containing medium ("standard medium") as described by Schwenkel and Winkelmann [18] and propagated by transfer to fresh standard medium every four weeks. Suspension cultures were established as described by Winkelmann et al. [19] and propagated by transfer to fresh standard medium every two weeks. Embryo development was induced by transfer of the cells to plant growth regulator-free medium [18], either growth regulator free standard medium or growth regulator free "U-medium" [65], which is a rich supplemented medium containing additional organic compounds and micro-elements.

**Table 2:** Description of tissues

tissue ID	cell line	culture	medium	developmental stage	embryogeneity	ploidy
1.2	F1 out of 3-2-0503	zygotic embryo	in planta	84-85 d after pollination	yes	diploid

1.3	F1 out of 3-2-0503	zygotic embryo	in planta	99-101 d after pollination	yes	diploid

2.1.1	3-14-0805	callus	standard	0 h before transfer to standard media without hormones	yes, but precocious germination	diploid

2.1.4	3-43-0503	callus	standard	0 h before transfer to standard media without hormones	no longer	tetraploid

2.1.6	3-43-0503	callus	standard without growth regulators	0 h before transfer to standard media without hormones	no longer	tetraploid

2.1.6B	3-45-0503	callus	standard without growth regulators	0 h before transfer to standard media without hormones	no longer	tetraploid

2.1.7	3-75-0503	suspension	standard	0 h before transfer to standard media without hormones	yes, but no torpedo-shaped s.e.	tetraploid

2.1.9	3-75-0503	suspension	standard without growth regulators	3d after transfer to standard media without hormones	yes, but no torpedo-shaped s.e.	tetraploid

2.1.10	3-76-0503	suspension	standard	0 h before transfer to standard media without hormones	yes, torpedo-shaped s.e.	tetraploid

2.1.11	3-76-0503	suspension	U without growth regulators	4 h after transfer to U media without hormones	yes, torpedo-shaped s.e.	tetraploid

2.1.12	3-76-0503	suspension	U without growth regulators	3d after transfer to U media without hormones	yes, torpedo-shaped s.e.	tetraploid

2.1.13	3-76-0503	somatic embryo out of suspension	U without growth regulators	20-22d - globular-shaped somatic embryo	yes, torpedo-shaped s.e.	tetraploid

2.1.14	3-76-0503	somatic embryo out of suspension	U without growth regulators	20-22d - torpedo-shaped somatic embryo	yes, torpedo-shaped s.e.	tetraploid

2.1.15	3-76-0503	somatic embryo out of suspension	U without growth regulators	20-22d - torpedo-shaped somatic embryo, precocious root germination	yes, torpedo-shaped s.e.	tetraploid

2.1.17	3-76-0503	somatic embryo out of suspension	U without growth regulators	20-22d - torpedo-shaped somatic embryo (following two step desiccation: 4d 97% and 3d 92% relative humidity)	yes, torpedo-shaped s.e.	tetraploid

2.3.1	3738-12G	callus	standard	0 h before transfer to standard media without hormones	never	diploid

2.3.3	3738-12G	callus	standard without growth regulators	3d after transfer to standard media without hormones	never	diploid

For RNA isolation cell material was collected 0 h, 4 h and 3 d after transfer to growth regulator-free medium. Selected globular- and torpedo-shaped embryos as well as torpedo-shaped embryos with roots were harvested 20 to 22 d after transfer to growth regulator-free medium. A part of the torpedo-shaped embryos was subjected to controlled slight desiccation over saturated salt solutions (4 d 97% relative humidity and 3 d 92% relative humidity, as described in Seyring and Hohe [23], prior to RNA isolation (for details see Table 2). In order to obtain zygotic embryos, cloned plants of genotype "3" were selfed and zygotic embryos were prepared out of the ovules 84 to 85 d after pollination for early stage torpedo-shaped embryos and 99 to 101 d after pollination for late stage torpedo-shaped embryos at the onset of desiccation. Due to spontaneous tetraploidisation of the embryogenic cell line during the study (Table 2), additional diploid and tetraploid callus lines were included when comparing zygotic and somatic embryos (Figure 1) in order to eventually reveal genes differentially expressed due to the ploidy status alone. Each analysed tissue was represented by three independent biological replicates.

### Microarray design

In a previous study, we generated an EST library from embryonic cell cultures of different developmental stages of *C. persicum *containing 1,980 assembled EST sequences [29]. Out of this, 1,216 annotated transcripts were used to generate a cDNA microarray (Additional file 1). *Escherichia coli *colonies containing the respective *C. persicum *cDNAs (> 0.5 kbp) cloned into pBluescript SK (+) were inoculated from glycerol cultures into overnight cultures in 96-well plates and plasmids were amplified from 1 μl culture using the Illustra TempliPhi Kit (GE Healthcare, München, Germany). The cDNA inserts were PCR-amplified in three 100-μl PCR reactions in GeneAmp 9600 thermocyclers (Perkin Elmer, Rodgau, Deutschland) with modified M13 primers complementary to 40 bp of the vector backbone of the cDNA clones (M13f-40, AGGGTTTTCCCAGTCACGACGTTGTAAAACGACGGCCAGT; M13r-40, TGTGAGCGGATAACAATTTCACACAGGAAACAGCTATGAC). The PCR products were cleaned and concentrated using Montage PCRμ96 Filter (Millipore, Schwalbach, Germany) and validated via conventional agarose gel electrophoresis. cDNAs of two human (factor IX (gi:183979970), vascular epithelial growth factor (gi:284172448)) and four *Saccharomyces cerevisiae *(YKL013c, YJR136c, YPL252C, YNL260C) transcripts were PCR-amplified to serve as heterologous negative control genes on the microarray. Each amplified cDNA was spotted four times onto NexterionE slides (Schott, Mainz, Germany) using an Omnigrid 100 arrayer (GeneMachines, CA, USA).

The Microaaray design has been desposited at ArrayExpress [66] and is accessible through the accession number E-TABM-837.

### RNA extraction and hybridisation

Total RNA was isolated from each biological replicate of the tissue samples according to the method described by Chang et al. [67] and purified using RNeasy mini columns (QIAGEN, Hilden, Germany). For microarray hybridisation we used a common reference design as proposed by Dudley et al. [68], in which a fluorescence labelled antisense oligonucleotide (complementary to a sequence tag present in all spotted microarray probes) is hybridised together with the labelled cDNA of interest. First strand cDNA was synthesised from 30 μg of total RNA using 100 Units Expand Reverse Transcriptase (Roche, Mannheim, Germany) and oligo-(dT) primer in the presence of 3 nmol Cy5-dUTP. Unincorporated nucleotides were removed using Microcon YM-30 (Millipore). Each labelled cDNA was pooled with 10.5 pmol Alexa555-labelled M13f-40 primer as antisense reference oligonucleotide and hybridised over night at 42°C to the microarray slide. The slides were washed three times successively in 2× SSC and 0.5% (w/v) SDS at 42°C, 0.5× SSC, and 0.1× SSC at room temperature for 5 min. The slides were scanned at 10 μm resolution with an ArrayWorXe scanner (Applied Precision, Washington, USA) and two-channel images were obtained for subsequent quantification of both Cy3 and Cy5 fluorescence intensities. Median pixel intensities of the spots were collected using GenePix Pro v. 6.1.0.2 (Molecular Devices, CA, USA). Local Background intensities were obtained from the median of all pixels within a defined area surrounding each spot (three times the diameter of the spot, excluding a three pixel margin surrounding any spot areas). Defective spots and areas were excluded manually.

### Analysis of gene expression data

Processing and statistical analysis of gene expression data was performed using Expressionist Pro v4.5 (Genedata, Basel, Switzerland), if not indicated differently. The quality of the expression data was assessed using the Refiner module of Expressionist. Only hybridisations were considered with less than three percent defective spots and yielding a quality of 96.5 or higher using the "Contrast" function of Refiner, which relies on signal-to-noise data. Hybridisations that did not fulfil these criteria were manually inspected in log-log plots of replicate arrays as well as by hierarchical clustering of the full data set and included in case of correct distribution of the data. Hybridisations were repeated until at least three microarrays passed this QC analysis for each analysed tissue. For tissues 1.2 and 2.1.7. data of four microarray slides was used. Background subtraction was performed using the Bayesian background subtraction method, which permits negative background-subtracted intensities (BSIs). Ratios between the BSIs from RNA and antisense oligonucleotide were calculated for each spot to obtain a measure of transcript abundance. Replicate spots per microarray slide were summarised by calculating the arithmetic mean (Microsoft Excel 2003). Summarised expression data of all arrays was subjected to Lowess normalization (f = 0.1) [69] to remove non-linearities in log-log plots of abundance values from different arrays. Differential gene expression was assessed using the regularised Bayesian unpaired t-test CyberT [70] and genes with p-values ≤ 0.005 were considered to be differentially expressed. Targets showing abundance values below the median of the heterologous negative controls in both conditions of the pairwise comparison were filtered out.

This data has been deposited at ArrayExpress [66] and is accessible through the ArrayExpress accession number E-TABM-837.

### Principal component analysis

Principal component analysis (PCA) was utilised to identify trends, clusters or outlying samples. PCA was performed on the normalised microarray data using the Expressionist Pro v5.1 software (Genedata, Basel, Switzerland) with genes as variables.

### Gene ontology annotation

Annotation of the *C. persicum *sequences using Gene Ontology and pathway mapping was carried out with the Blast2GO suite [71] and the KEGG Automatic Annotation Server [72]. The BLAST searches (BLASTX) were performed using the following parameter settings. Searches against Swiss-Prot (rel. 56) were performed with default parameters except that E-value cut-off was set to 1.0E-5. All sequences which did not yield a hit in the previous round were subjected to an additional search, with the same parameter settings, against the NCBI nr database (rel. 169). The annotation step was performed using the default parameters and followed by an InterProScan run. The resulting GO terms were merged and underwent the validation and augmentation step. The resulting GO annotation was mapped to GO slim terms using the Blast2GO internal mapping function with the goslim_tair.obo ontology set. It was tested which GO terms were significantly over- or underrepresented among the differentially expressed genes in the chosen experiments, as compared to the complete set on the chip. To test for significant bias, Fisher's exact test (p ≤ 0.05) was performed using the Expressionist 5.1 software (Genedata, Basel, Switzerland).

### Realtime PCR

Ten selected microarray results were validated by realtime PCR. For this purpose, the relative transcript abundance of homologues of a putative receptor kinase, two different GST, XET and/or POX (Table 3) were quantitatively measured in 3 different comparisons (Figure 3). PCR amplification was performed in a Stratagene Mx3000P realtime PCR System (Stratagene, La Jolla, CA, USA) using ABsolute QPCR SYBR Green ROX Mix (ABgene, Epsom, Surrey KT19 9AP, UK). First strand cDNA was synthesised from up to 1 μg of total RNA using QuantiTect^® ^Reverse transcription Kit (QIAGEN, Hilden, Germany). PCR reactions were carried out in a total volume of 25 μl, consisting of 2 ng cDNA, 400 nM forward primer, 400 nM reverse primer and 12.5 μl ABsolute QPCR SYBR Green ROX Mix. The realtime PCR program consisted of an initial denaturation step at 95°C for 15 min, 40 cycles of amplification with denaturation at 95°C for 15 sec, primer annealing for 1 min at 59°C and elongation at 72°C for 1 min. To complete the protocol, a melting range analysis with one cycle at 95°C for 1 min, 59°C for 1 min and 95°C for 30 sec with continuously measured fluorescence was performed. The reactions were performed in triplicate for each of three independent biological samples. All primer sequences are specified in Table 3. Standard curves were calculated for evaluating primer efficiency and all passed successfully.

**Table 3:** Primer sequences for realtime PCR

Name	Gene product	Forward primer (5'-3')	Reverse primer (5'-3')
CYC32T7_B02	Ef-Tu	CGCCATACTGCCTTTTTCTC	CTCCCGGCATAACCATCTTA

CYC01T7_E12	GST1	CATCCTGGGAGAACAATGTG	ACCCCCAAAGTAGGGTTTGT

CYC32T7_B11	GST2	GCTCGGGATTTTGCTAGAAG	TTCCCTGATGACAGAGCAAT

CYC04T7_G06	putative receptor kinase	CGTGGTGAGAGAAGAATGGA	GCATTTTAGGCCTCTTTTCG

CYC04T7_G04	POX	AAATCTCCAGCAAGGCAAAG	GCCGTGATAAAGGGACTGGTT

CP_59_C1	XET	TTCCGTGCAGGCTAAGTTCT	AGCGGAGCCTTCTGTATTGA

The values measured were normalised to the mean value of the reference gene (Ef-Tu) in each sample. The reference gene was selected because microarray results for this gene showed stable values over all tissues. The relative amount of PCR product generated from each primer set was determined on the basis of the cycle threshold (Ct) value. The relative quantity (RQ) was calculated by the ΔΔCt-method. The calculated relative quantity for one tissue is expressed as the ratio (fold change) to the tissue to which it was compared. If this number was less than one the (negative) reciprocal is given. The reported fold changes represent the arithmetic mean of the three independent experiments and three biological replicates. Differential gene expression was statistically assessed using a two-samplet-test (p ≤ 0.05).

### Histological analysis

Different stages of ovules, zygotic and somatic embryos were analysed anatomically. Materials were fixed in FAA solution containing 67% ethanol, 20% H_2_O, 1.8% formaldehyde and 5% glacial acetic acid for 24 h. The tissues were dehydrated by ethanol series and embedded in paraffine (J.T. Baker, Deventer, The Netherlands). Sections of 3-5 μm were prepared using a rotary microtome (RM 2155, Leica instruments, Nussloch, Germany). All samples were stained with FCA solution according to Etzold (New Fuchsine-Chrysoidine-Astra blue: 1000 ml dH_2_O, 0.1 g New Fuchsine, 0.143 g Chrysoidin, 1.25 g Astra blue and 20 ml glacial acetic acid) (Morphisto, Frankfurt, Germany). Pictures were taken using a light microscope (Zeiss, Axio Imager, Jena, Germany). Cell sizes were determined using the AxioVision v4.7.2.0 software (Carl Zeiss Imaging solutions, Jena, Germany). For each tissue 50 cells in 5 different samples were measured. Cell size of somatic embryos was analysed three weeks after induction, samples of zygotic embryos and of the endosperm were measured 60 days after pollination, i.e. before start of seed desiccation. Only diploid genotypes were used for cell size determination.

## List of abbreviations

AGO: Argonaute; Ef-Tu: elongation factor thermo unstable; GO: Gene Ontology; GST: glutathione S-transferase; SERK: somatic embryogenesis receptor kinase; PCA: principal component analysis; POX: peroxidase; s.e.: somatic embryogenesis; XET: xyloglucan endotransglycosylase.

## Authors' contributions

CH designed, performed and analysed the realtime PCR experiments and analysed the microarray results. SR planned and carried out the microarray experiments and evaluated their results. KK realised the histological experiments and carried out the tissue culture work. ADZ analysed the microarray data. AH conceived of and supervised part of the project and layed out the manuscript. SAR conceived of and supervised part of the project and analysed the microarray data. All authors contributed to writing of the manuscript and have seen and approved the final manuscript.

## Supplementary Material

Additional file 1 Annotation, fold changes and p-values of 1,216 transcripts present on the microarray Click here for file

Additional file 2 **Expression pattern of differentially expressed genes in the selected eight comparisons**. The symbol "+ -" in table indicates lower expression of a gene in the second tissue as compared to the first tissue of a comparison given in the column header. The symbol "- +" indicates higher expression of a gene in the second tissue as compared to the first tissue of a comparison given in the column header. Transcripts annotated as being homologous to genes previously described to be involved in somatic embryogenesis in other plants (NCBI Entrez query "somatic embryogenesis") are marked in turquoise and correspond to the annotation given in Additional file 1. Click here for file
